# Therapeutic effectiveness and safety of sintilimab-dominated triple therapy in unresectable hepatocellular carcinoma

**DOI:** 10.1038/s41598-021-98937-2

**Published:** 2021-10-05

**Authors:** Lei Dai, Xingchen Cai, Joseph Mugaanyi, Yelei Liu, Shuqi Mao, Changjiang Lu, Caide Lu

**Affiliations:** Department of Hepatopancreatobiliary Surgery, Ningbo Medical Centre Lihuili Hospital, Ningbo University, 1111 Jiangnan Road, Ningbo, 315040 Zhejiang China

**Keywords:** Cancer, Immunology

## Abstract

Immune checkpoint inhibitor therapy has shown promising results in patients with unresectable hepatocellular carcinoma. This study aimed to evaluate the effectiveness and safety of sintilimab, a programmed cell death protein-1 (PD-1) blockade, combined with sorafenib and transhepatic arterial chemotherapy and embolization in this patient population, compared with sintilimab monotherapy and sintilimab-sorafenib duotherapy. This was a 22 months single center retrospective cohort study in China. 80 patients with unresectable hepatocellular carcinoma were included, with diagnosis confirmed by either histologic, cytologic or diagnostic imaging analysis. The patients were divided into three groups based on therapeutic regimen: sintilimab monotherapy (sintilimab group, n = 22), sintilimab-sorafenib duotherapy (duplex group, n = 23), sintilimab-sorafenib and transcatheter arterial chemoembolization combined therapy (triple group, n = 35). The principal evaluation criteria were overall survival and progression free survival in the population, assessed according to response evaluation criteria in solid tumors, version 1.1 (RECIST 1.1). Secondary evaluation criteria were safety, objective response rate and disease control rate. From March 1st, 2019 to December 31, 2020, 80 patients with unresectable hepatocellular carcinoma were included and divided into three treatment groups (22 received sintilimab monotherapy, 23 received sintilimab-sorafenib duotherapy, and 35 received sintilimab-sorafenib combined with transcatheter arterial chemoembolization). The median overall survival of all patients was 11.0 months (95% CI 7.7–14.3). Median overall survival was 13.0 months (95% CI NE–NE), 9.0 months(95% CI 6.3–11.7)and 3.0 months (95% CI 1.9–4.1, *p* < 0.0001) in the triple therapy, duplex and sintilimab groups respectively, while the corresponding median progression-free survival were 5.0 months (95% CI 2.9–7.1, *p* < 0.001), 4.0 months (95% CI 2.8–5.2) and 2.0 months (95% CI 1.7–2.3). Disease control and clinical benefits rates were higher in the triple therapy group (80%, 95% CI 63.1–91.6, *p* < 0.001; 54.3%, 95% CI 36.6–71.2, *p* < 0.01) compared to the sintilimab group. Median duration of disease control was 4.0 months (95% CI NE–NE, *p* < 0.01) in the triple therapy group, longer than that of the duplex group (2.0 months, 95% CI 0.9–3.1) and sintilimab group (2.0 months, 95% CI 0.8–3.2). Grade 3 or 4 treatment-related adverse events occurred in 26.3% of 80 patients with hypertension was the most common event observed (38, 47.5%), however, other severe toxic effects were infrequent. Sintilimab combined with sorafenib and transcatheter arterial chemoembolization might have more beneficial effects on overall and progression-free survival and on the duration of disease control outcomes than both sintilimab monotherapy and sintilimab-sorafenib duotherapy in patients with unresectable hepatocellular carcinoma. This triple therapy model might represent an innovative and effective option for inoperable liver cancer.

## Introduction

Hepatocellular carcinoma (HCC) is one of the leading causes of cancer-related deaths worldwide^1^. There were more than 466,000 new cases of liver cancer and 422,000 liver cancer-related deaths in China in 2018^1,2^. Although early-stage liver cancer could be cured by surgical resection, liver transplantation, or radiofrequency ablation^3^, most patients with hepatocellular carcinoma are often diagnosed at an advanced stage and often present with distant metastasis or unresectable disease, amenable to neither surgery nor local therapy. Such patients are typically treated with systemic and translational therapies worldwide which have a poor prognosis: 10–18% 5-year overall survival^4–6^.

In the past decade, sorafenib, a multikinase inhibitor, was the only approved first-line systemic targeted agent for unresectable hepatocellular carcinoma^7,8^ while Oxaliplatin-based transarterial chemoembolization was approved as a standard systemic treatment in China^9^. However, both therapies have shown a poor objective response rate (2.0–8.2%) and overall survival benefit (6.4–10.7 months). Lenvatinib, an agent comparable to sorafenib in terms of overall survival (13.6 vs 12.3 months) was approved as a new first-line therapy option for patients with unresectable hepatocellular carcinoma^10^. Since 2017, regorafenib, nivolumab, cabozantinib, pembrolizumab, and ramucirumab have been approved successively for second-line therapy after sorafenib (objective response 4–17%, median overall survival 8.5–15.1 months)^11–15^. Despite the increase in options to prolong patient overall survival, there is still an urgent need to develop novel drugs and therapeutic models for unresectable hepatocellular carcinoma.

Programmed death protein-1/ligand-1 (PD-1/PD-L1) inhibitors have shown promising clinical prospects as potential treatment for hepatocellular carcinoma in phase 1–3 studies^12,15–17^. Nevertheless, they did not improve overall survival significantly in several phase 3 studies of single-agent therapy in first-/second-line settings, despite being associated with response rates in the 15–20% range^18,19^. Sintilimab is a highly selective fully human IgG4 monoclonal antibody against PD-1, that promotes the restoration of endogenous anti-tumor T cell responses by blocking the interaction between PD-1 and its ligands (PD-L1/2)^20^. It has been proven to provide encouraging clinical benefits in multiple solid tumor treatments as ether a monotherapy or combination therapy with acceptable and controllable toxicities^21–24^. Above all, the combination therapy with PD-1/PD-L1 analogues is more likely to be a promising and novel therapeutic option, such as anti-vascular endothelial growth factor (VEGF) enhancing anti-PD-1 and PD-L1 efficiency by reversing VEGF-mediated immunosuppression and promoting T-cell mediated tumor lysis^25,26^. Regardless of the high cost of treatment, the Atezolizumab-Bevacizumab combination, the latest globally acknowledged first-line standard treatment for advanced HCC, showed a significant benefit on objective response rate (ORR) (27.3%, [95% CI 22.5–32.5]) and overall survival (OS) at 12 months (67.2%, [95% CI 61.3–73.1])^17^. However, studies on the application of sintilimab monotherapy and combination therapy in unresectable hepatocellular carcinoma and the antitumor activity of PD-1/PD-L1-domiated multitherapy is minimal. This study therefore aimed to investigate the effectiveness and safety of sintilimab-dominated triple therapy which combined sintilimab, sorafenib and transcatheter arterial chemoembolization (TACE) in the patients with unresectable hepatocellular carcinoma.

## Methods

### Study design and patients

This was a retrospective cohort study that analyzed data of eligible patients (≥ 18 years) who received sintilimab monotherapy (sintilimab group), sintilimab-sorafenib duotherapy (duplex group), or sintilimab-sorafenib and TACE (triple group) at Ningbo University affiliated Lihuili hospital (eastern branch), Ningbo, from 1st March 2019 to 31st December 2020. There was no predetermined double-blind random allocation of these patients for receiving sintlimab monotherapy or combination therapy since the study was retrospective. All the patients were diagnosed with unresectable, locally advanced and/or metastatic hepatocellular carcinoma, which was confirmed by either histologic, cytologic or diagnostic imaging analysis according to the American Association for the Study of Liver Diseases criteria for patients with cirrhosis^27^.

The key inclusion criteria were as follows: (1) patients had not previously received systemic anti-tumor therapy and at least had one measurable lesion as defined by response evaluation criteria in solid tumors version 1.1 (RECIST 1.1), (2) a performance score of 0 to 1 on the Eastern Cooperative Oncology Group (ECOG) scale, (3) A to B classification on the Child–Pugh liver function scale, (4) a predicted life expectancy more than 3 months and adequate hematologic and organ function, (5) be tolerant and voluntary agree to the immune-treatments and signed informed consent of routine medical documents. (6) All the hepatitis B patients had received anti-hepatitis B therapy with entecavir for 1 year at least and had HBV-DNA index less than 3.00e + 1 IU/mL. Patients were excluded if they were at the end-stage of hepatocellular carcinoma, had a history of autoimmune disease, received locoregional treatment for HCC within 6 months prior to initial delivery, had previously received anti-PD-1 or anti-PD-L1 immunotherapy, and untreated or incompletely treated esophagogastric varices (assessed and treated with gastroscopy according to local clinical practice) with or at high risk of hemorrhage.

### Procedures

Patients group allocation was determined by the respective chief attending doctors based on the disease conditions (i.e. macrovascular invasion, extrahepatic metastasis, baseline of alpha-fetoprotein or Protein Induced by Vitamin K Absence or Antagonist-II (PIVKA-II) level, ECOG performance status, etc.) and patients’ willingness for therapy.

Patients in the sintilimab group were given 200 mg sintilimab intravenously over 30 min every 21 days. Patients in the duplex group were given 200 mg sintilimab intravenously over 30 min every 21 days plus 400 mg of sorafenib orally twice daily while patients in the triple group received sintilimab-sorafenib duotherapy accompanied with oxaliplatin (OXA, 60 mg/m^2^)–pirarubicin (THP, 20 mg/m^2^) emulsion TACE monthly for 4 cycles. Patients received their respective treatment until unacceptable toxic effects occurred or they were lost to follow-up due to death or gave up on treatment. If the unequivocal disease progression was absent, indicated by signs and symptoms and there was definite evidence of clinical benefit observed by investigators, patients could continue treatment beyond disease progression.

## Assessments

After the exclusion of a case of tumor thrombus detachment related pulmonary embolism death, data of 80 patients was extracted and included this study. Tumors were evaluated by Magnetic resonance imaging or computed tomography at baseline and subsequently every 9 weeks until treatment discontinuation. After disease progression or therapy discontinuation (whichever occurred later), the overall survival of patients was monitored every month until death, loss to follow-up, or end of study.

The primary performance used to analyze and compare the three regimens were: OS (defined as cumulative overall survival from the first treatment), progression free survival (PFS) (time from first treatment to initial radiological progression or death from any cause), disease control rate (DCR) (the percentage of patients whose best overall response was confirmed partial or complete response, or stable disease of at least 6 weeks), duration of disease control (DDC) (time from first response or stable disease to progression or death), ORR (the percentage of patients whose best overall response was confirmed partial or complete response), duration of objective response (DOR) (time from first response to progression or death) and clinical benefit rate (CBR) (the percentage of patients whose best overall response was confirmed partial or complete response, or stable disease of at 6 months). Besides, the univariate and multivariate analyses for the OS and PFS were performed.

Safety was continuously evaluated through vital signs and clinical laboratory test results including haematology, blood histochemistry, blood electrolyte, urinalysis, coagulation function and serum tumor markers concentration at the same frequency as imageology examination. In addition, the incidence and severity of adverse events were monitored and assessed from the first dose according to the National Cancer Institute Common Terminology criteria for adverse events version 4.0.

The patients’ characteristics, baseline data, comorbidities and operation history were collected at the start of the study. The protocol for this study was approved by the research ethics committee of Ningbo University affiliated Lihuili hospital (Approval number = KY2021PJ036), and conducted in accordance with the guidelines of the Declaration of Helsinki.

### Statistical analysis

Data are presented as mean ± standard deviation (SD). Effectiveness was assessed in all patients who were given any kind of treatment. Both OS and PFS were calculated and described by Kaplan–Meier analysis and the differences in parameters were compared among treatment groups using the stratified log-rank test as well as DOR and DCR. The 95% confidence interval (CI) was calculated using the Brookmeyer and Crowley method. The differences in ORR, DCR and CBR among the three groups were analyzed using Pearson’s chi-square test and the 95% CI calculated with the Clopper–Pearson method. The univariate and multivariate analyses for the OS and PFS were conducted by log-rank test or Cox regression Model respectively. Safety and adverse events in three groups were assessed and compared.

A *p* value < 0.05 was considered statistically significant. All analysis was performed with the Statistical Package for the Social Sciences (SPSS) version 26 (IBM, Armonk, NY, USA).

### Ethical approval

All procedures performed in studies involving human participants were in accordance with the ethical standards of the research ethics committee of Lihuili hospital affiliated to Ningbo University at which the studies were conducted (Approval no. KY2021PJ036) and with the 1964 Helsinki declaration and its later amendments or comparable ethical standards. Since this was an observational but not prospective intervention study, the Ethics Committee provided a waiver of informed consent.

## Results

### Baseline characteristics

Between March 1st, 2019 and December 31, 2020, 80 eligible patients with unresectable hepatocellular carcinoma were classified into three treatment groups (22 received sintilimab, 23 received sintilimab and sorafenib, and 35 received a combination of sintilimab, sorafenib and TACE). A flow diagram of the included and excluded patients was provided in the Fig. S1, and all the excluded patients received standard treatment based on clinical guidelines. The patients’ background, baseline characteristics, and medical history are summarized in Table 1. The average patient age was 55.2 ± 11.8 years and male patients (87.5%) were more common than female patients in this cohort study. 42.5% of patients received diagnosis based solely on radiology while 57.5% patients with a history of surgery were diagnoses and confirmed by histology and cytology. Each patient had a primary liver lesion with or without metastasis and/or vascular invasion. No other anti-tumor therapies were given during the follow-up period. There was no significant difference between the groups with respect to age, gender, Child–Pugh classification, Barcelona Clinic liver cancer stage, alcohol usage, hepatitis B virus infection, operation and ECOG performance status score. There was also no significant difference in laboratory data of the groups which shown in Table 1.

**Table 1:** Baseline characteristics of patients.

Variable	All treated patients	Sintilimab group	Duplex group	Triple group	*p* value*
	N = 80	N = 22	N = 23	N = 35	
Age (years)	55.2 ± 11.8	54.4 ± 10.8	54.0 ± 15.0	56.5 ± 10.2	ns
**Gender (no. (%))**					ns
Male	70 (87.5)	19 (86.4)	21 (91.3)	30 (85.7)	
Female	10 (12.5)	3 (13.6)	2 (8.7)	5 (14.3)	
**ECOG performance status score (no. (%))**					ns
0	43 (53.8)	11 (50.0)	12 (52.2)	20 (57.1)	
1	37 (46.3)	11 (50.0)	11 (47.8)	15 (42.9)	
**Child–Pugh classification (no. (%))**					ns
A	38 (47.5)	7 (31.8)	12 (52.2)	19 (54.3)	
B	42 (52.5)	15 (68.2)	11 (47.8)	16 (45.7)	
**BCLC stage (no. (%))**					ns
B	24 (30.0)	5 (22.7)	5 (21.7)	14 (40.0)	
C	56 (70.0)	17 (77.3)	18 (78.3)	21 (60.0)	
Alcohol use (no. (%))	13 (16.3)	4 (18.2)	6 (26.1)	3 (8.6)	ns
Hepatitis B virus infection (no. (%))	60 (75.0)	15 (68.2)	18 (78.3)	27 (77.1)	ns
**Concomitant disease (no. (%))**					
Hypertension	14 (17.5)	1 (4.5)	7 (30.4)	6 (17.1)	
Diabetes	10 (12.5)	1 (4.5)	3 (13.0)	6 (17.1)	
Gout	1 (1.3)	1 (4.5)	0 (0.0)	0 (0.0)	
Atrial fibrillation	1 (1.3)	0 (0.0)	0 (0.0)	1 (2.9)	
Operation (no. (%))	46 (57.5)	12 (54.5)	14 (60.9)	20 (57.1)	ns
**Microvascular invasion**					ns
0	5 (6.3)	1 (4.5)	3 (13.0)	1 (2.9)	
1	5 (6.3)	1 (4.5)	3 (13.0)	1 (2.9)	
2	4 (5.0)	0 (0.0)	2 (8.7)	2 (5.7)	
Extrahepatic metastasis	15 (18.8)	4 (18.2)	5 (21.7%)	6 (17.1)	ns
**Macrovascular invasion**					ns
Main portal vein	5 (6.3)	3 (13.6)	2 (8.7)	0 (0)	
Hepatic artery	40 (50.0)	10 (45.5)	13 (56.5)	17 (48.6)	
Both	2 (2.5)	1 (4.5)	1 (4.3)	0 (0)	
**Portal hypertension**					ns
None	39 (48.8%)	8 (36.4%)	12 (52.2%)	19 (54.3%)	
Mild	41 (51.3%)	14 (63.6)	11 (47.8%)	16 (45.7%)	
**Esophageal varices**					
None	70 (87.5%)	19 (86.4%)	20 (87.0%)	31 (88.6%)	ns
G1 (mild)	10 (12.5%)	3 (13.6%)	3 (13.0%)	4 (11.4%)	
Lg (α-fetoprotein) (µg/L)	2.5 ± 1.5	2.9 ± 1.6	2.1 ± 1.6	2.4 ± 1.4	ns
Lg (Des-γ-caboxy protein) (mAU/mL)	3.3 ± 1.3	3.8 ± 1.2	3.1 ± 1.4	3.1 ± 1.4	ns
Alanine aminotransferase (U/L)	59.0 ± 66.4	57.0 ± 45.2	41.3 ± 36.8	71.9 ± 87.8	ns
Aspartate aminotransferase (U/L)	78.8 ± 71.7	111.5 ± 106.0	57.2 ± 41.6	72.4 ± 53.0	ns
Total bilirubin (µmol/L)	34.8 ± 69.4	73.6 ± 124.3	21.2 ± 15.4	19.4 ± 12.8	ns
Prothrombin time (s)	13.3 ± 2.3	14.0 ± 2.9	13.6 ± 2.2	12.7 ± 1.8	ns
International normalized ratio	1.2 ± 0.2	1.2 ± 0.2	1.2 ± 0.2	1.1 ± 0.2	ns

Data are presented as n (%) or mean ± SD.

α-fetoprotein and Des-γ-caboxy protein were calculated by means of log (− log) transformation.

*ns* not significant, *BCLC* Barcelona Clinic liver cancer.

*Compared with each group (one-way ANOVA test, or Pearson’s chi-square test).

### Effectiveness

**Figure 1:**
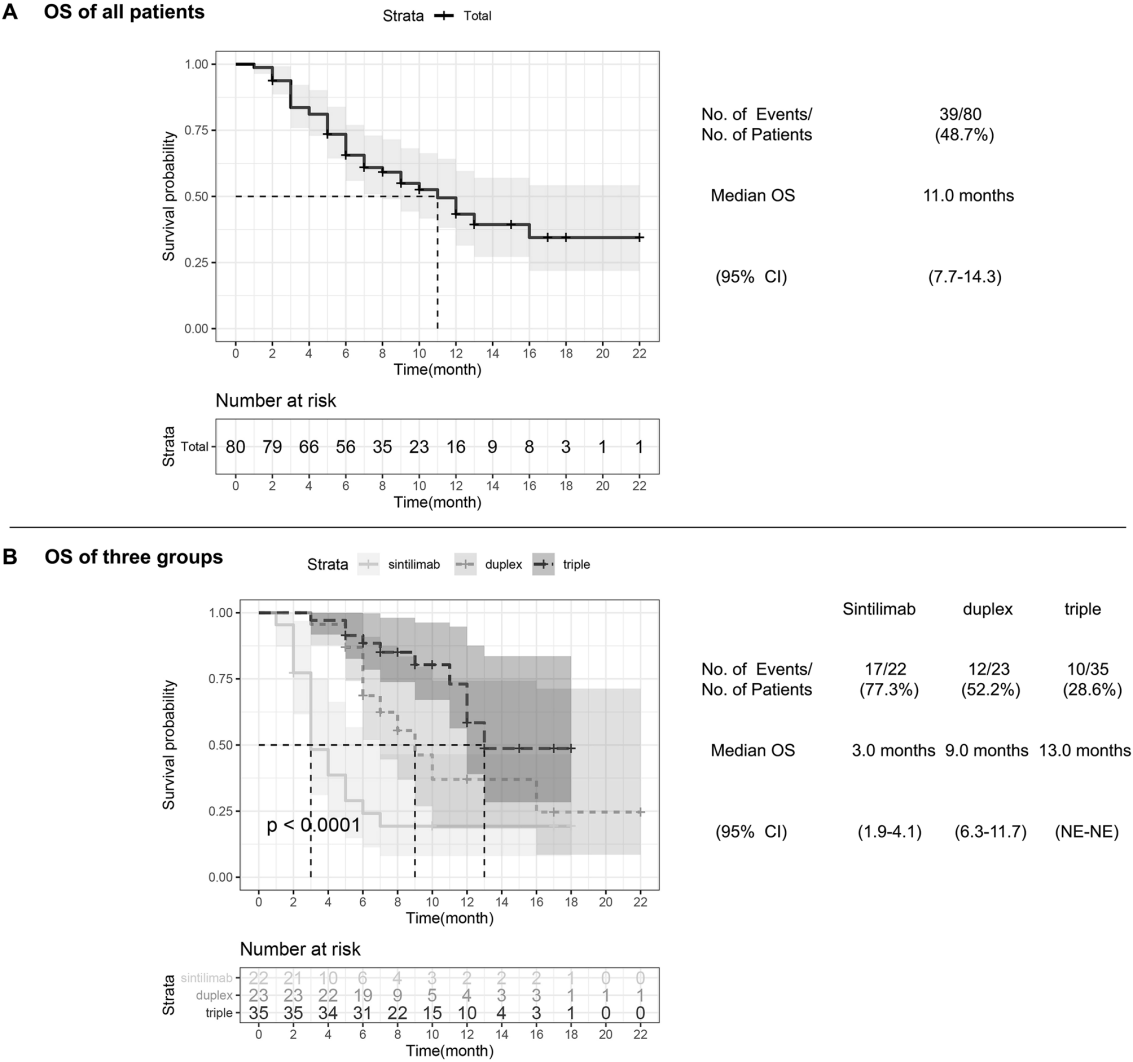
Kaplan–Meier analysis of overall survival. (**A**) All patients’ Kaplan–Meier analysis of overall survival (OS) are shown, according to response evaluation criteria in solid tumors, version 1.1. Median OS is 11.0 months (95% CI 7.7–14.3). (**B**) Kaplan–Meier estimates of OS among sintillimab, duplex and triple groups are shown. *p*-value calculated is < 0.0001. CI denotes confidence interval, and NE could not be evaluated.

As of the date of clinical data collection cutoff, December 31, 2020, the median OS of all patients was 11.0 months (95% CI 7.7–14.3), with a total of 39 patient (48.7%) death at the end of follow-up (Fig. 1A). The median OS of the triple group was 13.0 months (95% CI NE–NE), which was longest of the three treatment groups with a mortality rate of 28.6% (*p* < 0.0001) (Fig. 1B). In comparison, the sintilimab group had an OS of 3.0 months (95% CI 1.9–4.1) and a mortality rate of 77.3% while the OS of the duplex group was 9.0 months (95% CI 6.3–11.7) which was significantly longer (*p* = 0.005) (Table 2) than the former. The triple group’s OS was in turn significantly longer than that of the duplex group (*p* = 0.040).

**Table 2:** The multiple comparison of between-group variance of survival analysis.

	Sintilimab vs duplex		Sintilimab vs triple		Duplex vs triple	
	χ^2^	*p* value*	χ^2^	*p* value*	χ^2^	*p* value*
OS	7.80	0.005	22.29	< 0.001	4.21	0.040
PFS	6.86	0.009	11.96	0.001	0.57	0.450
DOR	0.04	0.850	3.90	0.048	2.67	0.103
DDC	0.13	0.722	4.00	0.045	5.14	0.023
ORR	1.09	0.297	1.71	0.191	0.04	0.836
DCR	8.01	0.005	13.23	< 0.001	0.30	0.587
CBR	2.72	0.099	9.42	0.002	2.12	0.145

*OS* overall survival, *PFS* progression free survival, *DOR* duration of object response, *DDC* duration of disease control, *ORR* object response rate, *DCR* disease control rate, *CBR* clinical benefit rate.

*Compared with each group (Kaplan–Meier Analysis or Pearson’s chi-square test), *p* value < 0.05 was considered statistically significant.

**Figure 2:**
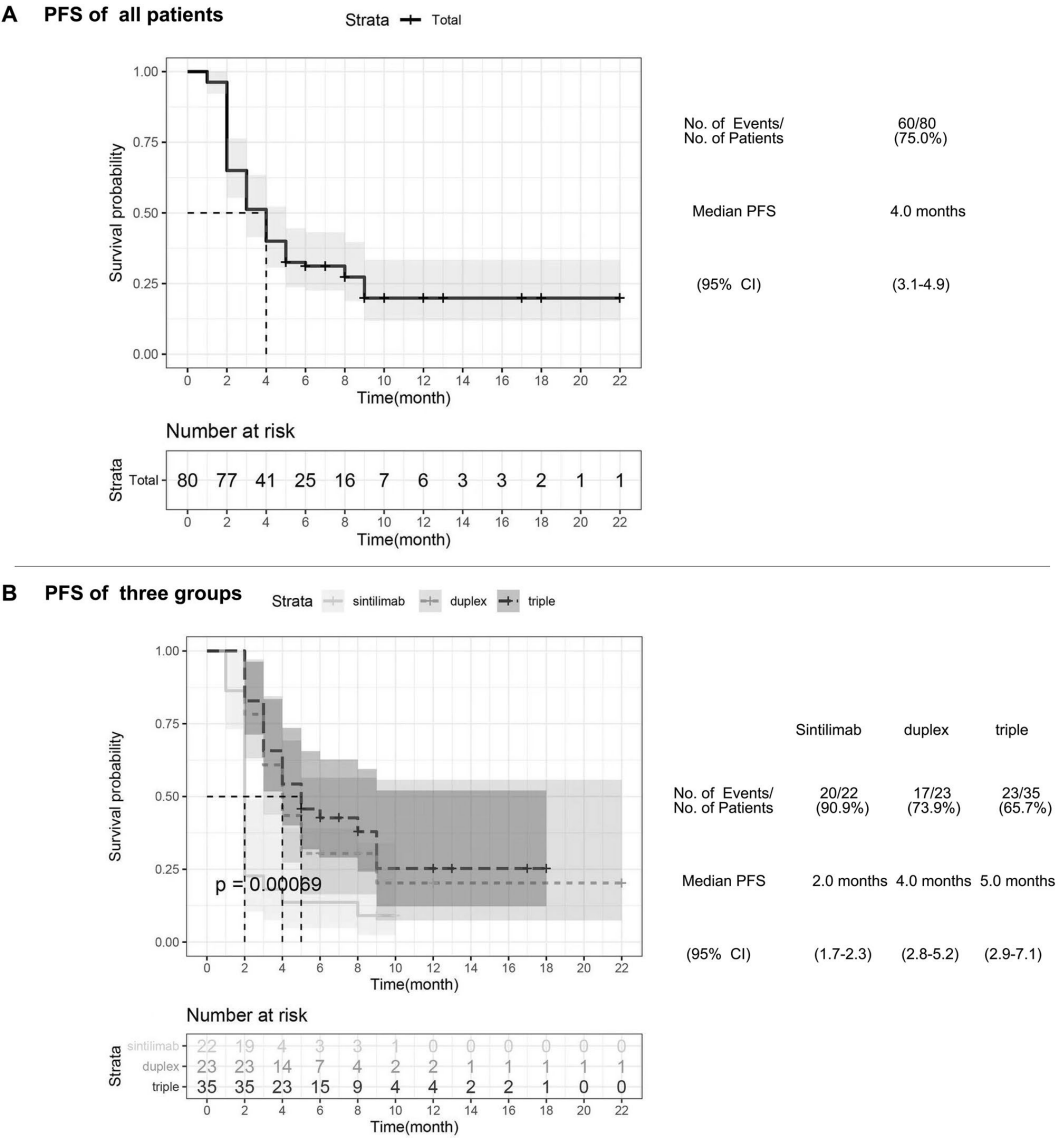
Kaplan–Meier analysis of progression free survival. (**A**) All patients’ Kaplan–Meier analysis of progression free survival (PFS) are shown, according to response evaluation criteria in solid tumors, version 1.1. Median PFS is 4.0 months (95% CI 3.1–4.9). (**B**) Kaplan–Meier estimates of PFS among sintillimab, duplex and triple groups are shown. *p*-value calculated is 0.00069. CI denotes confidence interval.

Disease progression or death was observed in 60 patients (75.0%) across all three treatment regimens with a median PFS of 4.0 months (95% CI 3.1–4.9) (Fig. 2A). Median PFS of the triple group and the duplex group were significantly longer than that of the sintilimab group (5.0 months, [95% CI 2.9–7.1] and 4.0 months, [95% CI 2.8–5.2] respectively vs 2.0 months, [95% CI 1.7–2.3], *p* < 0.001) (Fig. 2B). However, there was no statistically difference between the duplex and triple group (*p* = 0.450) (Table 2).

Regarding the secondary assessment criteria, the best overall responses per group are shown in Table 3. Most patients in each group were in a stable disease (SD) state (33, 41.3%). 12 (15%) of the 80 patients achieved complete response (CR), half of which were given sintilimab combined sorafenib and TACE. On the contrary, 15 (68.2%) of 22 patients in the sintilimab group achieved progressive disease (PD). The CR, partial response (PR), SD and PD ratios were (17.4% vs 17.1%), (8.7% vs 11.4%), (47.8% vs 51.4%) and (26.1% vs 20%) respectively for the duplex group vs triple group (*p* > 0.05).

**Table 3:** The comparison of tumor responses in three groups.

	All treated patients	Sintilimab group	Duplex group	Triple group	*p* value*
	N = 80	N = 22	N = 23	N = 35	
**Best overall response**					0.020
CR	12 (15.0%)	2 (9.1%)	4 (17.4%)	6 (17.1%)	0.660
PR	7 (8.8%)	1 (4.5%)	2 (8.7%)	4 (11.4%)	0.670
SD	33 (41.3%)	4 (18.2%)	11 (47.8%)	18 (51.4%)	0.034
PD	28 (35.0%)	15 (68.2%)	6 (26.1%)	7 (20%)	0.001
ORR	19 (23.8%)	3 (13.6%)	6 (26.1%)	10 (28.6%)	0.415
(95% CI)	(14.9–34.6)	(2.9–34.9)	(10.2–48.4)	(14.6–46.3)	
DCR	52 (65.0%)	7 (31.8%)	17 (73.9%)	28 (80.0%)	0.001
(95% CI)	(53.5–75.3)	(13.9–54.9)	(51.6–89.8)	(63.1–91.6)	
CBR	30 (37.5%)	3 (13.6%)	8 (34.8%)	19 (54.3%)	0.008
(95% CI)	(26.9–49.0)	(2.9–34.9)	(16.4–57.3)	(36.6–71.2)	

Data are presented as n (%, 95% CI) or n (%).

*CR* complete response, *PR* partial response, *SD* stable disease, *PD* progressive disease, *ORR* object response rate, *DCR* disease control rate, *CBR* clinical benefit rate.

*Compared with each group (Pearson’s chi-square test).

Given that both the OS and PFS results were statistically significant, objective response rates, disease control rates and clinical benefit rates were sequentially compared (Table 3). According to independent assessment with RECIST 1.1, the confirmed objective response rates were 13.6% (95% CI 2.9–34.9) with sintilimab monotherapy, 26.1% (95% CI 10.2–48.4) with sintilimab-sorafenib and 28.6% (95% CI 14.6–46.3) with sintilimab combined sorafenib and TACE (*p* = 0.415). CR ratio (*p* = 0.660) and PR (*p* = 0.670) had no statistical difference among the three groups however differences in SD (*p* = 0.034) and PD (*p* = 0.001) were significant. The disease control rates (objective response plus stable disease) were 31.8% (95% CI 13.9–54.9), 73.9% (95% CI 51.6–89.8), and 80% (95% CI 63.1–91.6) respectively in the sintilimab monotherapy, duplex and triple therapy group (*p* = 0.001). Furthermore, there was a statistically significant difference in clinical benefit rates among the three groups (*p* = 0.008); 13.6% (95% CI 2.9–34.9), 34.8% (95% CI 16.4–57.3) and 54.3% (95% CI 36.6–71.2) for the sintilimab monotherapy, duplex and triple groups respectively. The triple group was superior to both the sintilimab group (*p* < 0.008) and the duplex group (*p* = 0.005) in regards to DDC, however, there was no significant difference between the duplex and the triple group (*p* = 0.587). Similarly, the CBR of the triple group was much considerably higher than that of the sintilimab group (*p* = 0.002), although there was no clear superiority of the duplex group over the sintilimab group (*p* = 0.099), nor the triple group over to the duplex group (*p* = 0.145).

**Figure 3:**
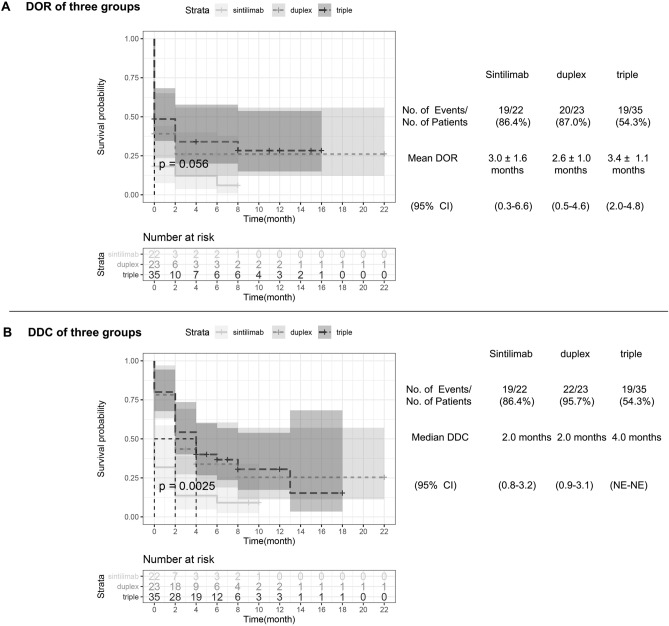
Kaplan–Meier analysis of duration of object response and duration of disease control. (**A**) Kaplan–Meier estimates of duration of object response (DOR) among sintillimab, duplex and triple groups are shown, according to response evaluation criteria in solid tumors, version 1.1. Mean DOR are presented as mean ± SD, *p* value calculated is 0.056. (**B**) Kaplan–Meier estimates of duration of disease control (DDC) among sintillimab, duplex and triple groups are shown. *p*-value calculated is 0.0025. CI denotes confidence interval, and NE could not be evaluated.

The calculated mean duration of objective response of the sintilimab group was 3.5 ± 1.6 months (95% CI 0.3–6.6), longer than that of the duplex group (2.6 ± 1.0 months, [95% CI 0.5–4.6]) and the triple group (3.4 ± 1.1 months, [95% CI 2.0–4.8]), nevertheless there was no statistical significance (*p* = 0.056) (Fig. 3A). The estimated median duration of disease control was 2.0 months in the sintilimab group (95% CI 0.8–3.2) and the duplex group (95% CI 0.9–3.1), which were shorter than in the triple group (4.0 months, [95% CI NE–NE], *p* = 0.0025) (Fig. 3B).

Additionally, we included all variables but therapy methods in the univariate analysis of the OS and PFS. ECOG (*p* = 0.014), Child–pugh classification (*p* = 0.001), portal hypertension (*p* = 0.004), esophageal varices (*p* = 0.034), AST index (*p* = 0.001), TB index (*p* < 0.001), PT index (*p* < 0.001), INR index (*p* < 0.001) and Macrovascular invasion (*p* = 0.027) were discovered to be associated with OS significantly, so as the microvascular invasion (*p* = 0.013), TB index (*p* = 0.001), PT index (*p* = 0.010) and INR index (*p* = 0.022) to PFS (Table 4). Furthermore, these factors above were included into the multivariate analysis of OS and PFS. According to the Table 5, we found that AST index (HR 1.006, 95% CI [1.001, 1.011], *p* = 0.011) and PT index (HR 1.231, 95% CI [1.100, 1.377], *p* < 0.001) were independent factors affecting the OS of patients with unresectable hepatocarcinoma. TB index (HR 1.005, 95% CI [1.002, 1.008], *p* = 0.003) was an independent factor impacting the PFS of hepatocarcinoma patients.

**Table 4:** The univariate analysis for the OS and PFS.

	OS		PFS	
	χ^2^	*p* value*	χ^2^	*p* value*
Age	0.144	0.704	1.315	0.252
Gender	0.001	0.972	0.254	0.614
ECOG	6.073	**0.014**	0.719	0.396
Child–Pugh	10.123	**0.001**	3.370	0.066
BCLC	1.870	0.171	0.110	0.740
Alcohol use	0.165	0.684	0.146	0.702
HBV infection	1.528	0.216	0.273	0.601
operation	1.117	0.291	0.072	0.789
MVI	4.589	0.205	10.804	**0.013**
Extrahepatic metastasis	1.648	0.199	2.624	0.105
Co-diseases	10.913	0.053	4.749	0.447
Macrovascular invasion	9.216	**0.027**	2.101	0.552
AFP index	0.638	0.424	0.521	0.471
DCP index	2.105	0.147	0.668	0.414
Portal hypertension	8.214	**0.004**	2.390	0.122
Esophageal varices	4.508	**0.034**	1.764	0.184
ALT index	0.167	0.683	0.381	0.537
AST index	10.709	**0.001**	3.341	0.068
TB index	18.508	** < 0.001**	10.556	**0.001**
PT index	18.795	** < 0.001**	6.719	**0.010**
INR index	16.379	** < 0.001**	5.223	**0.022**

*OS* overall survival, *PFS* progression free survival, *BCLC* Barcelona Clinic liver cancer, *MVI* microvascular invasion.

*Compared with each group (Log-Rank test or Omnibus test for univariate). *p* < 0.05 means statistically significant (highlighted in bold).

**Table 5:** The multivariate analysis for the OS and PFS.

	OS			PFS		
	HR	95% CI	*p* value*	HR	95% CI	*p* value*
ECOG	NE	NE	0.476	NE	NE	0.722
Child–pugh	NE	NE	0.110	NE	NE	0.257
MVI	NE	NE	0.526	NE	NE	0.350
Macrovascular invasion	NE	NE	0.949	NE	NE	0.863
Portal hypertension	NE	NE	0.227	NE	NE	0.386
Esophageal varices	NE	NE	0.501	NE	NE	0.977
AST index	1.006	1.001–1.011	**0.011**	NE	NE	0.241
TB index	NE	NE	0.140	1.005	1.002–1.008	**0.003**
PT index	1.231	1.100–1.377	** < 0.001**	NE	NE	0.079
INR index	NE	NE	0.520	NE	NE	0.143

*OS* overall survival, *PFS* progression free survival, *MVI* microvascular invasion, *NE* could not be evaluated.

*Compared with each group (Cox regression analysis with adjusted hazard). *p* < 0.05 means statistically significant (highlighted in bold).

At the end of follow-up, we performed survival and disease progression analysis for all participants. 39 (48.7%) of 80 participants had died due to disease progression. Causes of death were: 1 pulmonary embolism death, 1 due to pulmonary metastasis related hemoptysis, 2 heart failures, 35 cases of liver decompensation, including 26 cases of jaundice, 5 cases of refractory ascites and 4 cases of hepatic encephalopathy. 41 (51.3%) were still alive and 34 of whom were progression-free. 7 developed liver decompensation, including 4 with ascites and 3 with jaundice.

### Safety

A total of 80 patients who received sintilimab treatment (monotherapy or in combination with other therapies) were involved in safety analysis. Treatment-related adverse events of any grade are summarized in Table 6. The most common treatment-related adverse events of any grade were hypertension in 38 (47.5%) of 80 patients, fatigue in 19 (23.8%), diarrhea in 16 (20.1%) and abnormal liver function in 16 (20.1%). Grade 3 or 4 adverse events were infrequent compared to Grade 1 and 2, the most common of which were hypertension (8, [10%]) and liver dysfunction (4, [5%]). Hypertension had the highest incidence of treatment-related adverse events in the three groups: 11 (49.9%) of 22, 12 (52.1%) of 23 and 15 (42.8%) of 35 in the sintilimab, duplex and triple groups respectively.

**Table 6:** Treatment-related adverse events.

	All patients N = 80			Sintilimab group N = 22			Duplex group N = 23			Triple group N = 35		
	Grade1	Grade2	Grade3–4	Grade1	Grade2	Grade3–4	Grade1	Grade2	Grade3–4	Grade1	Grade2	Grade3–4
Hypertension	18 (22.5%)	12 (15%)	8 (10%)	5 (22.7%)	3 (13.6%)	3 (13.6%)	6 (26.1%)	5 (21.7%)	1 (4.3%)	7 (20.0%)	4 (11.4%)	4 (11.4%)
Diarrhea	13 (16.3%)	0	3 (3.8%)	3 (13.6%)	0	1 (4.5%)	2 (8.7%)	0	0	8 (22.9%)	0	2 (5.7%)
Fatigue	19 (23.8%)	0	0	5 (22.7%)	0	0	7 (30.4%)	0	0	7 (20.0%)	0	0
Pyrexia	9 (11.3%)	1 (1.3%)	2 (2.5%)	3 (13.6%)	1 (4.5%)	0	1 (13.0%)	0	0	3 (8.6%)	0	2 (5.7%)
Abnormal liver function	11 (13.8%)	1 (1.3%)	4 (5%)	3 (13.6%)	0	0	5 (21.7%)	0	0	3 (8.6%)	1 (2.9%)	4 (11.4%)
Constipation	12 (15%)	0	0	2 (9.1)	0	0	3 (13.0%)	0	2 (8.7%)	5 (14.3%)	0	0
Blood bilirubin increase	7 (8.8%)	2 (2.5%)	2 (2.5%)	1 (4.5%)	0	0	2 (8.7%)	1 (4.3%)	2 (8.7%)	4 (11.4%)	1 (2.9%)	0
Rash	8 (10%)	2 (2.5%)	2 (2.5%)	1 (4.5%)	1 (4.5%)	1 (4.5%)	4 (17.4%)	0	0	3 (8.6%)	1 (2.9%)	1 (2.9%)
Abnominal pain	6 (7.5%)	0	0	3 (13.6%)	0	0	1 (4.3%)	0	0	2 (5.7%)	0	0
Epistaxis	2 (2.5%)	0	0	1 (4.5%)	0	0	1 (4.3%)	0	0	0	0	0
PS	5 (6.3%)	4 (5%)	0	2 (9.1%)	0	2 (9.1%)	1 (4.3%)	0	2 (8.7%)	2 (5.7%)	0	0
Cough	3 (3.8%)	0	0	1 (4.5%)	0	0	1 (4.3%)	0	0	1 (2.9%)	0	0

Data are presented as n (%).

*PS* Palmar-planter erythrodys esthesia syndrome.

Disease progression aside, there were no treatment adverse event related deaths or discontinuations. All the adverse events took a favorable turn after symptomatic and supportive treatment. Serious adverse events were more frequent in the sintilimab dominated triple therapy group (13, [37.1%]) than in the duplex (7, [30.4%]) and monotherapy groups (7, [31.7%]). Except for hypertension, the most common grade 3 or 4 adverse event in the triple group was liver dysfunction (4, [11.4%]). Constipation (2, [8.7%]) and elevated blood bilirubin (2, [8.7%]) were the most common grade 3 or 4 adverse events in the duplex group. In contrast, diarrhea (1, [4.5%]), rash (1, [4.5%]), and Palmar-Planter Erythrodysesthesia (2, [9.1%]) were the most common grade 3 or 4 adverse events in the sintilimab group.

No significant differences in the incidence of grade 1 treatment-related adverse events were observed among the three groups. There was also an observed phenomenon that a patient would present with several adverse events simultaneously or sequentially, although they were tolerable.

## Discussion

This single-center retrospective study is the first research of immunotherapy (sintilimab)-dominated multiple treatment for Chinese patients with unresectable hepatocellular carcinoma so far. The results showed statistically significant improvements in overall survival, progression-free survival and duration of disease control when sintilimab was given in combination with sorafenib and TACE than when administered as a sintilimab-sorafenib duotherapy or as a sintilimab monotherapy in unresectable hepatocellular carcinoma patients without previous systemic treatment.

Although sintilimab has not been approved for hepatocellular carcinoma first-line therapy in china, it has been approved for classical Hodgkin’s lymphoma in patients who have relapsed or are refractory after 2 or more lines of systemic chemotherapy^28^ and nonsquamous non-small cell lung cancer (NsqNSCLC)^21^. It showed more effectiveness with better progression-free survival and comparable safety to camrelizumab and toripalimab in an hepatitis B virus related hepatocellular carcinoma cohort study^24^.

More than 70% of hepatocellular carcinoma patients in China have HBV infection, whereas the majority of patients with hepatocellular carcinoma in the USA, Europe and Japan have HCV infections^29^. It is consistent with the finding of a 75% HBV infection rate in our study (Table.1). Although the KEYNOTE-224^12^ and CheckMate 040^15^ studies showed that the effectiveness of nivolumab and pembrolizumab was not related to HBV or HCV infection, further validation of such findings is still needed due to their small sample size. As reported in prior studies, pembrolizumab^18^ (62.2% of DCR) in the second-line setting after sorafenib, nivolumab^30^ (55% of DCR) and camrelizumab^16^ (46.8% of DCR) in sorafenib-experienced patients showed considerable curative effect. These results might suggest that HCC patients treated with Tyrosine kinase inhibitor (TKI) developed certain changes that could synergistically enhance anti-tumor activity of PD-1 inhibitors. Sintilimab-dominated duotherapy (73.9% of DCR) and triple therapy (80% of DCR) showed relatively higher disease control rates than sintilimab monotherapy (31.8% of DCR) (*p* = 0.001) in our study, indicating that double- or multi-agent therapy dominated by immunotherapy agents is more effective than single-immunotherapy agent strategy.

However, the low proportion of DCR in the sintilimab monotherapy group (31.8%), which might be due to the poorer baseline clinical characteristics reported in the patients in our study, including higher proportions of patients with an ECOG performance status score of 1, Child–Pugh classification of B, high level of Total bilirubin and Aspartate aminotransferase, necessitate further randomized controlled prospective studies to reduce bias and interferences. Although the objective response rate of the sintilimab monotherapy group (13.6%) was relatively lower, potentially contributing to the low proportion of CR, PR and SD, sintilimab combined with sorafenib (26.1%) or sorafenib-TACE (28.6%) achieved a much higher ORR. However, there were no statistically significant differences among the three groups (*p* = 0.415). Above all, we observed a higher clinical benefit rate with sintilimab combined sorafenib and TACEthan sintilimab combined sorafenib or as a monotherapy, 54.3% vs 34.8% vs 13.6% (*p* = 0.008), which has rarely been reported before about PD-1 inhibitors.

According to the previous studies, pembrolizumab and nivolumab might prolong the PFS of advanced HCC patients to 5.5 months (95% CI 3.5–7.4) and 4.6 months (95% CI 3.0–6.2) respectively^31^, indicating a promising effectiveness of PD-1 inhibitors on improving HCC patient survival. In contrast, we observed a relatively shorter overall PFS (4.0 months, [95% CI 3.1–4.9], which may be attributable to the small sample and retrospective trial bias. There was a similar result of median OS between our study and other studies (11.0 months, [95% CI 7.7–14.3] vs 11.0 months, [95% CI 8.2–13.8] with pembrolizumab or nivolumab), although the proportion of BCLC stage C is much higher in our study accompanied with shorter follow-ups. In addition, several therapeutic effects between subgroups were evaluated. Except the duration of objective response, which there was not statistically significant among the three treatment groups (*p* = 0.056), sintilimab combined with sorafenib and TACE was much longer than sintilimab-sorafenib duotherapy and sintilimab monotherapy in regards to median OS (*p* < 0.0001), median PFS (*p* = 0.00069) and median DDC (*p* = 0.0025). These results suggest that sintilimab-dominated comprehensive treatment combined with sorafenib and TACE might achieve longer OS, PFS and DDC and increased benefit for patients with unresectable hepatocellular carcinoma. In general terms, the triple therapy was significantly better than monotherapy in terms of therapeutic effectiveness in the least. On the other hand, it indicated that immunotherapy-dominated triple or multiple treatment might create a new therapy model for hepatocellular carcinoma, even for other solid tumors, if tolerable by patients.

Overall, sintilimab had a safety profile similar to other anti-PD-1 agents, except for the occurrence of hypertension. Although nearly half of patients presented with any grade of hypertension (47.5%), it was clinically controllable and could be improved by treatment and most manifested with mild symptoms. Similar to the molecular mechanism of camrelizumab which is most frequently characterized by reactive cutaneous capillary endothelial proliferation^32,33^, the binding epitope of sintilimab is different from that of other PD-1 immune checkpoint inhibitors. We hypothesize that the reactivation of the immune response by sintilimab may play a key role in interrupting some unknown signalling pathway in the pathogenesis of hypertension. However, this idea will need to be proven by further investigation in future studies. Owing to the small sample size and shortness of follow-up of our study, some immune-related adverse events such as sintilimab-induced autoimmune diabetes^34^ and myocarditis^35^ might have not been observed. Another common adverse event was liver dysfunction (11.5% with grade 1–2 and 11.4% with grade 3–4) in the triple group, which might be due to a synergistic hepatotoxicity from sorafenib and TACE. Other treatment-related adverse events were mild or moderate in the three groups indicating acceptable safety of sintilimab.

PD-L1 positivity has been reported to be associated with longer PFS in patients who receive atezolizumab-bevacizumab combination therapy than in patients treated with sunitinib^36^. However the predictive value of PD-1 status and tumor mutational burden has not been presented in the case of hepatocellular carcinoma^17,19^. Genetic or blood-based biomarker analyses (or both) will need to be conducted in future to identify relevant biomarkers of response and that can be used to screen the patients who would benefit most from PD-1-dominated multiple therapy.

There are several limitations to take into account for this study. First, it was a retrospective cohort study in nature consisting of three groups with a small sample size in a single center. Since there were no indications and protocols for appropriate candidate inclusion, several terminal-stage patients were included in the sintilimab monotherapy group. Therefore, selection bias may have some baring on the results of this study. A high proportion of patients with Child–Pugh B were included, which probably influenced the patients’ survival and hypothesis validity. The outcomes of our study need to be further confirmed with a large, multicenter, open-label, randomized, prospective trial to evaluate the long-term efficiency and safety of sintilmab-dominated triple therapy in patients with unresectable hepatocellular carcinoma. Although sintilimab combined sorafenib and TACE may prolong the OS, PFS and DDC of patients with unresectable hepatocellular carcinoma to a certain extent, the study was short-term with a short follow-up, hence some of the patients were still under follow at the end of the study. Despite being a 22 months retrospective cohort study, our results also demonstrated a better prognosis and safety of sintilimab-dominated triple therapy for patients with unresectable hepatocellular carcinoma compared to duotherapy and monotherapy. However, whether sintilimab-dominated multiple therapy is superior to monotherapy or other anti-PD-1/PD-L1 agent needs to be prospectively evaluated. Due to insufficient data, we were not able to include a control group receiving standard of care (Sorafenib) for advanced HCC. Although Lenvatinib (had been demonstrated not to be inferior to sorafenib), PD-1 (Pembrolizumab or Nivolumab) and PD-L1 (Atezolizumab) through several clinical trials have been suggested to be of potential benefit to HCC patients, at the time of our study, Sorafenib was still the standard first-line therapy. Regardless of the challenges to Sorafenib’s usage recommendations, we were convinced that comparison of sintilimab and Sorafenib monotherapy was more scientific and logical. To address the limitations resulting for a non-controlled study, further controlled studies are warranted to further evaluate our findings of improved effectiveness with sintilimab-dominated combination therapy.

In conclusion, the results of this study indicate that sintilimab-dominated immunotherapy combined with sorafenib and TACE could enhance the anti-tumor activity of single-agent therapy and potentially improve preliminary survival. Determination of the regimen’s efficacy and safety profile in patients with unresectable hepatocellular carcinoma required further prospective studies.

## Supplementary Information

Supplementary Legends.

Supplementary Figure 1.
