# Supplementary material for: Therapeutic effectiveness and safety of sintilimab-dominated triple therapy in unresectable hepatocellular carcinoma
Source: Sci Rep. 2021 Oct 5;11:19711. doi: 10.1038/s41598-021-98937-2 (PMC8492645; doi:10.1038/s41598-021-98937-2)
Supplement: Supplementary file 1 — Supplementary Legends. [file 41598_2021_98937_MOESM1_ESM.docx]

**Figure S1. Flow diagram outlining the study’s patient inclusion-exclusion criteria.** HCC, hepatocellular carcinoma. TACE, transcatheter arterial chemoembolization.
