# Supplementary figures and images for: Therapeutic effectiveness and safety of sintilimab-dominated triple therapy in unresectable hepatocellular carcinoma
Source: Sci Rep. 2021 Oct 5;11:19711. doi: 10.1038/s41598-021-98937-2 (PMC8492645; doi:10.1038/s41598-021-98937-2)

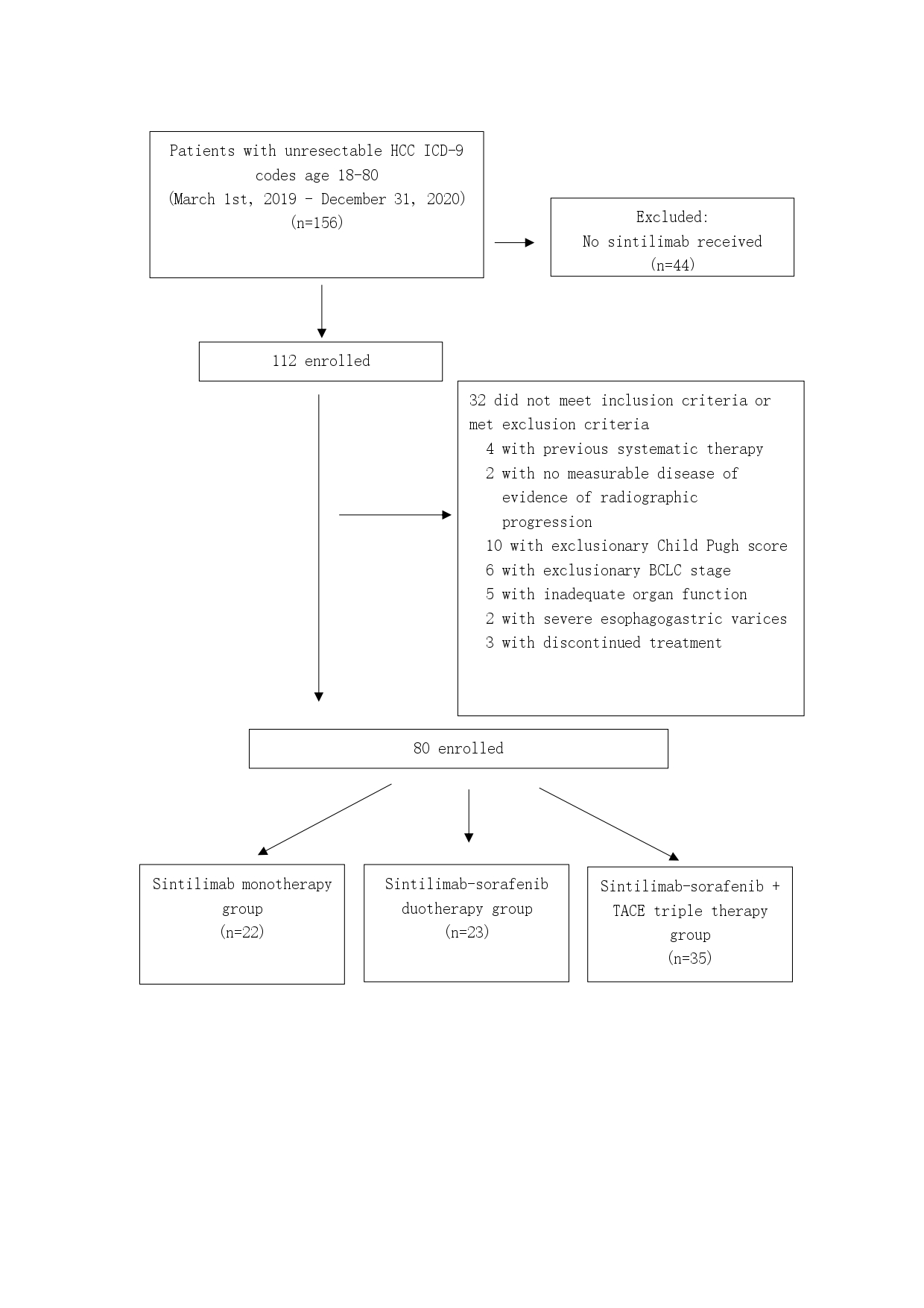

Supplement: Supplementary file 2 — Supplementary Figure 1. [file 41598_2021_98937_MOESM2_ESM.jpg]
